# Treatment of Abnormal Vaginal Microbiota before Frozen Embryo Transfer: Case-Report and Minireview to Discuss the Longitudinal Treatment Efficacy of Oral Clindamycin

**DOI:** 10.3389/fphys.2017.00415

**Published:** 2017-06-19

**Authors:** Thor Haahr, Helle O. Elbaek, Rita J. Laursen, Birgit Alsbjerg, Jørgen S. Jensen, Peter Humaidan

**Affiliations:** ^1^Department of Clinical Medicine, Denmark and the Fertility Clinic Skive, Skive Regional Hospital, Aarhus UniversitySkive, Denmark; ^2^Microbiology and Infection Control, Statens Serum InstitutCopenhagen, Denmark

**Keywords:** vaginal microbiota, clindamycin, frozen embryo transfer, bacterial vaginosis

## Abstract

Abnormal vaginal microbiota (AVM) or bacterial vaginosis (BV) might negatively impact reproductive outcomes of *in vitro* fertilization (IVF). However, before randomized controlled trials are initiated to investigate cause and effect, it is necessary to establish the optimal treatment for AVM. Metronidazole seems ineffective to treat the biofilm in AVM; thus, clindamycin could be suggested as a relevant antibiotic agent for future intervention based studies. In the present case report, we present the first longitudinal follow-up of the vaginal microbiota with molecular methods during and after oral clindamycin treatment. Furthermore, we review the recent literature with the aim to discuss the optimal AVM treatment in a fertility setting. The patient was 40 years old suffering from unexplained secondary infertility. Prior to the present transfer cycle, she had had two failed IVF cycles. The tentative explanation of failed treatment was age-related aneuploidy. However, the patient asked for AVM diagnosis and she was subsequently diagnosed and treated successfully. Unfortunately, the patient did not achieve pregnancy after clindamycin treatment and two subsequent frozen embryo transfer cycles. Taken together, we report an excellent AVM treatment efficacy both short-term and long-term following oral clindamycin treatment. We discuss the potential impact on the vaginal microbiota of co-treatment with estrogen patches in the stimulated frozen embryo transfer cycle. Furthermore, we discuss future aspects of AVM treatment such as the potential impact of estrogen and live biotherapeutic products to positively modulate the microbiota of the reproductive tract.

## Introduction

During the last two or three decades, the diagnostic approach to BV has been either the clinical Amsel Criteria (pH > 4.5, fishy odor, clue cells on wet smear, vaginal discharge) or different scoring methods of Gram stained vaginal smears, e.g., Nugent score or the Ison-Hay criteria (Amsel et al., 1983; Nugent et al., 1991; Ison and Hay, 2002). Today, molecular based techniques that targets typical BV bacteria such as *G. vaginalis* and *A. vaginae* have been suggested to provide superior performance compared to the Gram stained approach, mainly due to better bacterial specificity, more objective reading, no microscopy time for the clinician and, importantly, the advantage of a yes/no diagnosis (Menard et al., 2010; Datcu et al., 2013). However, the use of quantitative PCR (qPCR) assays or next generation sequencing diagnostic methods are still in limited clinical use. One major reason being the lack of consensus for defining abnormal vaginal microbiota (AVM) by a molecular based diagnosis compared to BV. Furthermore, the causal relation between molecularly defined AVM and different reproductive outcomes are still at an investigational stage. On the other hand, BV has been suggested to be a risk factor for obstetric complications such as miscarriage and preterm birth (Hay et al., 1994; Ralph et al., 1999; Donders et al., 2000) but the clinical impact of treatment for BV has been difficult to prove (Haahr et al., 2016a). Consequently, BV is rarely screened for and treated unless symptomatic, and this might only involve 20% of BV cases (Koumans et al., 2007).

The prevalence of BV has been reported to be higher among infertile patients compared to pregnant patients in ethnically comparable Danish cohorts, 21 and 14%, respectively (Thorsen et al., 2006; Haahr et al., 2016b). A recent study from our group suggested that AVM may be associated with poor reproductive outcomes in IVF patients (Haahr et al., 2016b). The pathogenesis could be that *G.vaginalis* and *A.vaginae* ascend from the vagina to the endometrium to establish infection and biofilm formation that negatively impacts the implantation of the embryo (Swidsinski et al., 2013). This hypothesis has been corroborated by a recent study of the endometrial microbiota of IVF patients, in which a *Lactobacillus* species (spp.) depleted endometrial microbiota was significantly associated with poor reproductive outcomes (Moreno et al., 2016). Taken together, much research is needed to further understand the complex interplay between the implanting embryo and the microbiota of the reproductive tract. To further investigate causation, intervention based studies are urgently needed and in that regard, an important question is: how do we modulate the vaginal microbiota from unfavorable to favorable?

When considering the close similarity between BV and AVM, the standard treatment regimens for BV, metronidazole and clindamycin, seem to be the first line choice for AVM treatment. However, both *in vitro* and *in vivo* studies have reported that metronidazole was unable to effectively eradicate BV associated bacteria and especially the biofilm of BV (Nagaraja, 2008; Swidsinski et al., 2008). Thus, clindamycin could be suggested as the most promising antibiotic for a future randomized controlled trial (RCT) on treatment of AVM. To our knowledge, no data has been presented to document the longitudinal treatment response of oral clindamycin administered for molecularly diagnosed AVM in an IVF setting.

Here, we present a case-report documenting the longitudinal effect of oral clindamycin on AVM and discuss our findings in the context of the available literature.

## Case-report

**Figure 1:**
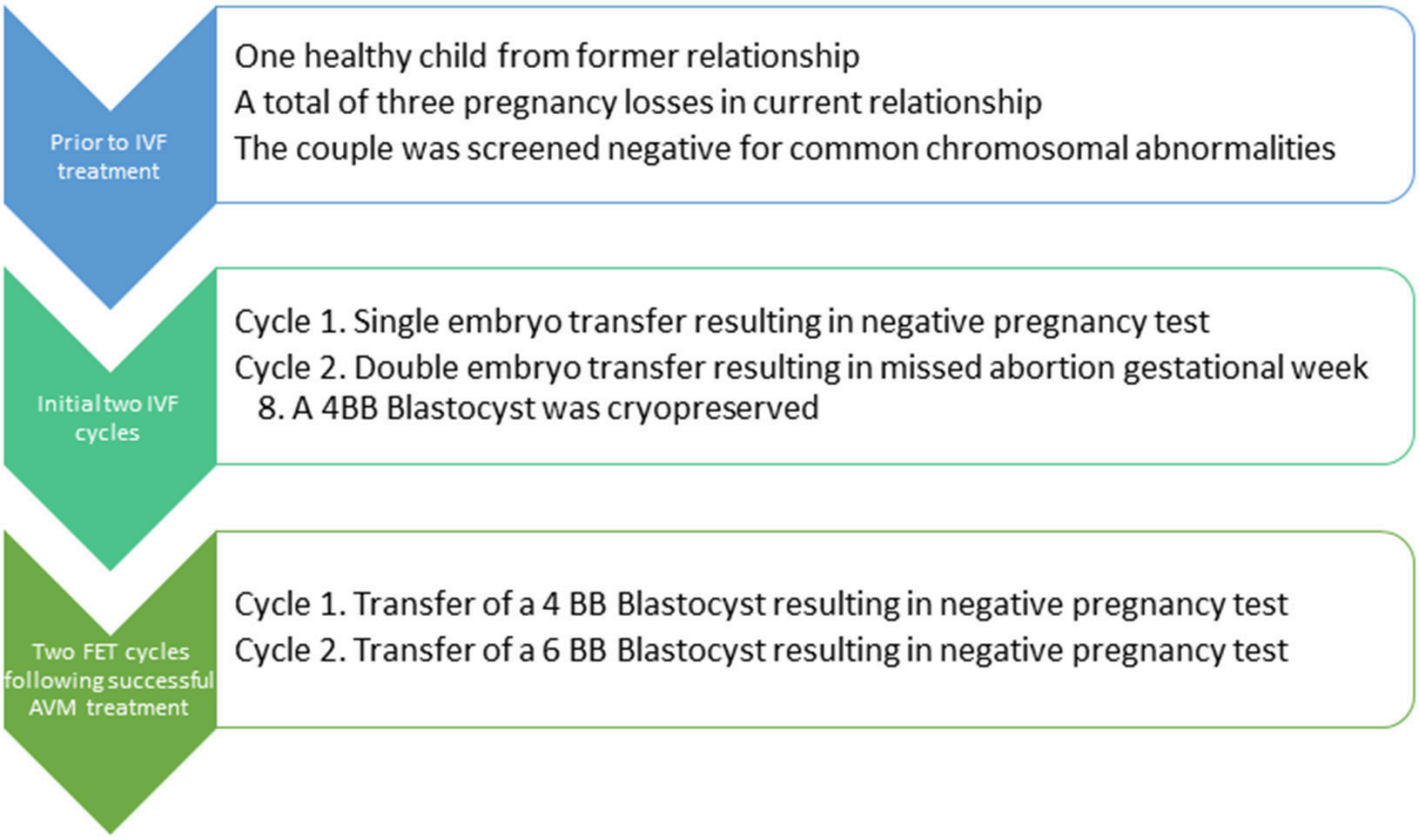
Flowchart for the patient. IVF, *In vitro* fertilization; FET, Frozen embryo transfer; AVM, Abnormal vaginal microbiota.

In the present case-report, we discuss the clinical management of a couple attending their first frozen embryo transfer (FET) cycle following two failed fresh IVF cycles, see flowchart in Figure 1. At entry to our clinic, the patient was 39 years old and had one child with a previous partner following a natural conception and after an uncomplicated pregnancy. In the present relationship, she had a history of one early spontaneous abortion (not ultrasound verified), one missed abortion (gemelli) and one late miscarriage at 22 gestational weeks (Trisomy 16). The pregnancy losses were all from natural conceptions. The patient had a body mass index of 21, she was a non-smoker and reported no use of alcohol. Previously, the patient was diagnosed with myxedema which was well-treated with 100 μg of levothyroxine throughout the study period. Her husband had normal semen quality and the couple had no chromosomal abnormalities in a screening examination. Due to Danish law, pre-implantation genetic screening (PGS) of embryos could not be performed.

After the two failed fresh IVF cycles and considering the history of recurrent pregnancy loss (Figure 1), a hysteroscopy was performed where endometrial adhesions were excised. Then, after consultation of the couple, a stimulated FET cycle was initiated, i.e., estrogen stimulation was started for preparation of the endometrium to embryo transfer (ET). On her own initiative, the patient was tested for *Ureaplasma* spp. by the general practitioner who found her to be negative for *Ureaplasma* spp. Despite no reported BV symptoms, the patient was worried about BV and the potential impact on her fecundability. Consequently, the vaginal pH was measured and a vaginal swab was obtained for AVM diagnosis using quantitative (q) threshold assessment of *G. vaginalis* and *A. vaginae* by qPCR (Datcu et al., 2013; Haahr et al., 2016b). The pH was 4.0 and in a vaginal smear which underwent Nugent score assessment, the patient was graded with normal vaginal flora. However, in the qPCR analysis, the vaginal swab sample was found to be positive for *G. vaginalis* above threshold, but negative for *A. vaginae*. It was explained to the patient, that the evidence for a positive impact on the chance of pregnancy by treatment was poor. However, due to patient demand, she was treated with oral clindamycin for 7 days, 300 mg two times per day.

The patient was instructed to take self-collected swabs (Copan Eswab) from the mid-vagina and a follow-up swab was collected at the day of embryo transfer to test the short-term treatment efficacy 8 days after last clindamycin dose. Long term treatment efficacy was tested 4 months later. The patient reported no side-effects from clindamycin during the treatment period until the follow-up consultation 8-days later.

**Figure 2:**
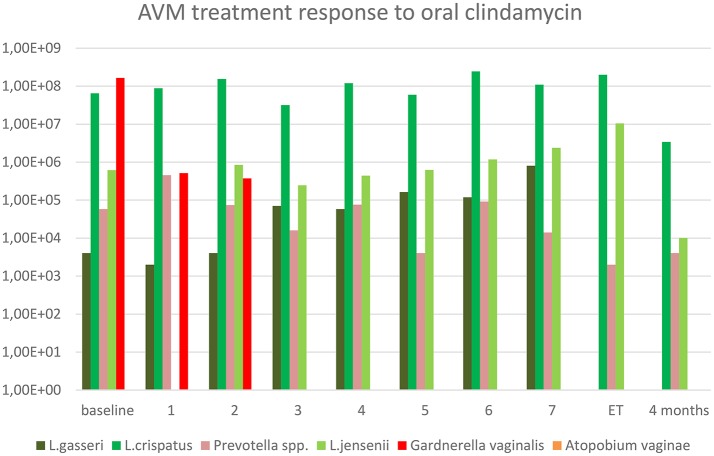
Treatment of abnormal vaginal microbiota for 7 days with clindamycin in an IVF patient. Y-axis represents copy numbers (copies/mL) from qPCR analysis. X-axis represents the sampling time, i.e., 1 means day 1 after first clindamycin intake. ET, Embryo transfer at 8 days follow-up.

As shown in Figure 2, the short- and long-term treatment efficacy from oral clindamycin on AVM was excellent resulting in rapid eradication of *G. vaginalis*. Interestingly, the clindamycin treatment did not seem to significantly affect the *L. crispatus* load in this patient. By molecular testing, the patient was negative for *Candida* spp. throughout the study period.

Along with the treatment for AVM, the patient was routinely treated with exogenous estrogen in a step-up regimen to support the endometrial growth prior to FET. Estrogen patches (Vivelle Dot® were administered from 100 μg on first stimulation day and subsequently increased to 400 μg resulting in an endometrial thickness of 7 mm at FET 34 days later. The 4BB blastocyst from stimulation number two was transferred. However, the hCG pregnancy test was negative on day 14. The tentative explanation for the failed cycles was age-related aneuploidy as less than 40% of embryos will be euploid in a 40-year old woman (Franasiak et al., 2014). Therefore, a third stimulation cycle was performed aiming for segmentation with a downregulation protocol with GnRH agonist in an attempt to maximize the oocyte pool. A total of 7 oocytes were retrieved, resulting in one 6BB blastocyst for cryo-preservation. A follow-up vaginal swab was collected on the first day of stimulation and this swab was without detectable *G. vaginalis* and the patient continued to FET with the 6BB blastocyst. Unfortunately, the hCG test was negative on day 14. Despite of good treatment results of AVM, other contributing factors might have led to the negative pregnancy test; however, aneuploidy was by far the most likely factor.

The regional ethical committee of Central Region Denmark stated that ethical approval was not necessary, as this case was part of clinical management. Furthermore, the patient gave written consent to the clinical management and subsequent publication.

## Discussion and review of the literature

To the best of our knowledge this case-report is the first to investigate the longitudinal effect of treatment with oral clindamycin on the vaginal microbiota by qPCR method throughout the treatment course and with a substantial follow-up. The effect on *G.vaginalis* was striking, with total eradication within 3 days, but surprisingly, very little effect was seen in the load of *L. crispatus* (Figure 1).

Both clindamycin and metronidazole are considered standard treatments for BV. We chose to treat this patient with clindamycin and not metronidazole as it has been reported that a large proportion of *G. vaginalis* strains are resistant to metronidazole *in vitro* (Nagaraja, 2008) and due to the fact that metronidazole did not effectively eradicate the BV biofilm *in vivo* (Swidsinski et al., 2008). Furthermore, the effect of BV-treatment on adverse obstetrical outcomes was only shown to be significant for clindamycin treatment as reported in recent clinical recommendations concerning BV in pregnancy (Haahr et al., 2016a). Clindamycin was given orally instead of topically because of the low systemic concentrations after topical therapy and thus, theoretically, a low concentration in the endometrium where a *G.vaginalis* biofilm was previously documented (Levinson et al., 2005; Swidsinski et al., 2013). In contrast, a major drawback of clindamycin as compared to metronidazole is the susceptibility of *Lactobacillus* spp. to clindamycin with MIC90 (the minimal antimicrobial concentration that will inhibit 90% of all strains) for *L. crispatus* < 4 μg/ml (Melkumyan et al., 2015; Petrina et al., 2016). The observation that *L. crispatus* levels were unaffected during the antibiotic treatment period was, therefore, highly unexpected. One explanation could be that the particular *L. crispatus* strain colonizing this patient had a higher MIC than usual, but this is contradicted by the substantial growth in the last part of the treatment cycle of *L. jensenii*, pointing more in the direction of a sub-inhibitory concentration of clindamycin in some parts of the vaginal compartment as it would be unlikely that two different species should have unusually high MICs. This patient did not have BV according to conventional Nugent criteria, however, when we observed the high *G. vaginalis* load in qPCR, we found scientific basis to investigate the treatment response. The patient was informed about the limited evidence for a positive treatment effect, but she preferred to enter the case-study and undergo treatment. She had a low vaginal pH and already in the pretreatment sample, she had a high *L. crispatus* bacterial load. The low pH may have influenced the activity of clindamycin in the vaginal compartment, as reported for *Staphylococcus aureus*, the clindamycin MIC increased 4-fold by a decrease in pH from 7.4 to 5.5 (Lemaire et al., 2011). As the endometrial pH is ~7 regardless of the bacterial dominance (Moreno et al., 2016), it could be speculated that oral clindamycin had sufficient activity in the uterine cavity to eradicate the *G. vaginalis* biofilm and apparently also vaginal *G. vaginalis*, yet, leaving the vaginal lactobacilli unaffected. Whether the same sparing of *Lactobacillus* spp. would occur in the higher pH environment found in true BV remains to be determined. Another important positive modulator of *Lactobacillus* spp. could be the co-treatment with intensive estrogenic support, which was E2 patches in the present case. In a recent report on Chinese menopausal women treated with low dose oral estrogens for atrophic vaginitis, it was reported that increasing levels of serum estradiol increased the abundance of *Lactobacillus spp*. in a dose response manner, whereas the relative abundance of *G. vaginalis* and other anaerobes decreased (Shen et al., 2016)—an effect that potentially could have impacted the results in the present case-report, see Figure 2. Finally, we might have overemphasized the potential role of *G.vaginalis* in AVM dysbiosis, as it might not cause adverse effects alone. Although we investigated selected common vaginal bacteria, we did not report data using sequencing techniques that would have shown a more comprehensive overview of the vaginal microbiome. On the other hand, the presence of *G.vaginalis* even in relatively high absolute concentrations in the vaginal fluid may easily go undetected by 16S rRNA gene microbiota studies in the presence of a high load of lactobacilli or other bacteria as this technique provide data on the relative concentration. This is in contrast to the very low limit of detection by qPCR and the absolute concentration measurement provided by this technique.

Regarding the future aspects, it should be considered which infertile patients could potentially benefit from iatrogenic modulation of the vaginal microbiota prior to ET. We have previously shown that AVM was associated with a poor reproductive outcome in IVF patients (Haahr et al., 2016b). These results should lead to a RCT that investigates the potential impact of AVM treatment on the reproductive outcome. However, before future RCT's can be conducted there is a need to investigate the optimal treatment for AVM. In the present case, we present a case that illustrates that oral clindamycin for 7 days seemed to modulate the vaginal microbiota in a very beneficial way. However, as discussed above, it could be suggested that the treatment effect was not due to clindamycin alone considering the contribution of intensive co-treatment with estrogenic patches. Another upcoming adjuvant to or maybe even stand-alone treatment of AVM, is live biotherapeutic products (LBPs) or probiotics (Ross et al., 2008; Hemmerling et al., 2010; Larsson et al., 2011). However, strain documentation and manufacturing issues call upon well-conducted RCTs with strain specific LBPs to adequately assess the treatment effect of these new drugs. Finally, we envision that future AVM treatment studies could investigate the potential impact in recurrence rate and also clinical outcomes of male/partner treatment, which is currently not standard of care.

## Concluding remarks

In the present case, *G. vaginalis* was successfully eradicated after oral clindamycin in a woman undergoing IVF treatment, however, pre-existing *Lactobacillus* spp. were unaffected. This finding may have important implications for the selection of the optimal route of administration of clindamycin in treatment trials of AVM in women preparing for IVF treatment, as AVM has been shown to be negatively associated with reproductive outcomes following IVF.

## Author contributions

TH, JJ, BA, and PH contributed to study design and manuscript drafting. HE, PH, TH, RL, JJ, and BA were part of the clinical management. All authors contributed to, revised and accepted the final manuscript.

### Conflict of interest statement

TH has received an honorarium from Bifodan A/S. PH received unrestricted research grants from MSD, Merck, and Ferring as well as honoraria for lectures from MSD, Merck, and IBSA. PH, TH, and JJ received a research grant from Osel Inc. The other authors declare that the research was conducted in the absence of any commercial or financial relationships that could be construed as a potential conflict of interest.
